# COMP-angiopoietin-1 ameliorates inflammation-induced lymphangiogenesis in dextran sulfate sodium (DSS)-induced colitis model

**DOI:** 10.1007/s00109-018-1633-x

**Published:** 2018-04-02

**Authors:** Ae Sin Lee, Mi Jeong Sung, Won Kim, Yu Jin Jung

**Affiliations:** 1 Korea Food Research Institute, 245, Nongsaengmyeong-ro, Iseo-myeon, Wanju_Gun, Jeollabuk-do 55365 Republic of Korea; 2 Department of Internal Medicine, Division of Nephrology, Chonbuk National University Medical School, Jeonju, Republic of Korea; 3 Research Institute of Clinical Medicine of Chonbuk National University, Biomedical Research Institute of Chonbuk National University Hospital, Jeonju, Republic of Korea

**Keywords:** Angiopoietin-1, Lymphangiogenesis, Inflammation, Macrophages, Colitis, VEGF

## Abstract

**Abstract:**

Alterations in the intestinal lymphatic network are pathological processes as related to inflammatory bowel disease (IBD). In this study, we demonstrated that reduction in inflammation-induced lymphangiogenesis ameliorates experimental acute colitis. A soluble and stable angiopoietin-1 (Ang1) variant, COMP-Ang1, possesses anti-inflammatory and angiogenic effects. We investigated the effects of COMP-Ang1 on an experimental colonic inflammation model. Experimental colitis was induced in mice by administering 3% dextran sulfate sodium (DSS) via drinking water. We determined body weight, disease activity indices, histopathological scores, lymphatic density, anti-ER-HR3 staining, and the expression of members of the vascular endothelial growth factor (VEGF) family and various inflammatory cytokines in the mice. The density of lymphatic vessel endothelial hyaluronan receptor 1 (LYVE-1) and VEGFR-3-positive lymphatic vessels increased in mice with DSS-induced colitis. We observed that COMP-Ang1-treated mice showed less weight loss, fewer clinical signs of colitis, and longer colons than Ade-DSS-treated mice. COMP-Ang1 also significantly reduced the density of LYVE-1-positive lymphatic vessels and the disruption of colonic architecture that is normally associated with colitis and repressed the immunoregulatory response. Further, COMP-Ang1 treatment reduced both M1 and M2 macrophage infiltration into the inflamed colon, which involved inhibition of VEGF-C and D expression. Thus, COMP-Ang1, which acts by reducing inflammation-induced lymphangiogenesis, may be used as a novel therapeutic for the treatment of IBD and other inflammatory diseases.

**Key messages:**

- COMP-Ang1 decreases inflammatory-induced lymphangiogenesis in experimental acute colitis.
- COMP-Ang1 improves the symptom of DSS-induced inflammatory response.
- COMP-Ang1 reduces the expression of pro-inflammatory cytokines in inflamed colon.
- COMP-Ang1 reduces the expression of VEGFs in inflamed colon.
- COMP-Ang1 prevents infiltration of macrophages in a DSS-induced colitis model.

**Electronic supplementary material:**

The online version of this article (10.1007/s00109-018-1633-x) contains supplementary material, which is available to authorized users.

## Introduction

Ulcerative colitis (UC) is an inflammatory disease that affects the colon and the small intestine. Clinical diagnosis revealed hematochezia, passage of mucus, distorted crypt architecture, and crypt abscesses in patients with UC [1]. Numerous animal models of colonic inflammation with several features of UC exist, which require administration of specific concentrations of colitis-inducing chemicals such as dextran sulfate sodium (DSS) [2].

Inflammation induces inflammatory lymphangiogenesis, and remodeling of lymphatic vessels in inflamed conditions is important for tissue homeostasis and immune response [3]. In fact, lymphatic vessel density in the inflamed colonic mucosa of patients with UC increases with progression of the disease [4]. Reports show that blockade of angiogenesis, the growth of new blood vessels, could be a new therapeutic approach in experimental colitis models [5].

Angiopoietin-1(Ang1) was identified as a secreted protein ligand of tyrosine kinase with important roles in vascular development [6, 7]. Ang1 possesses anti-inflammatory effect and reduces vascular permeability [8]. The N-terminal portion of Ang1 was replaced with the short coiled-coil domain of cartilage oligomeric matrix protein (COMP) to generate COMP-angiopoietin-1, a soluble, stable, and potent Ang1 variant [9]. Previous studies show that COMP-angiopoietin-1 (COMP-Ang1) affects wound healing by enhancing angiogenesis and lymphangiogenesis in a diabetic experimental model [10]. Furthermore, blood serum angiopoietin-1 levels are elevated in patients with UC and can be used as a factor for studying the progression of inflammatory bowel disease (IBD) [11, 12].

Macrophages are essential for the pathogenesis of IBD. Inflammatory macrophages in the colon act as initiators and protectors of immune responses of IBD-related disorders of the epithelial barrier [13, 14]. Polarized macrophage phenotype is classified into two functional types, namely, classically activated macrophages (M1) and alternatively activated macrophages (M2) [15]. M1 macrophages secrete pro-inflammatory cytokines and contribute to the initiation of DSS-colitis, along with innate immune cells, neutrophils, and dendritic cells. On the contrary, M2 macrophages produce anti-inflammatory cytokines, which exhibit protective roles in the development of IBD [16].

In this study, we investigated the mechanism underlying the effect of COMP-Ang1 on colitis symptoms and changes in lymphatic vessel density in acute colitis. The effect of COMP-Ang1 on activated macrophages in colitis was also investigated to determine whether they are involved in the colonic immune response.

## Materials and methods

### Animal experiments

Seven-week-old male C57BL/6 mice (Charles River Korea, Seoul, South Korea) were used as experimental animals. All animal studies were reviewed and approved by the Institutional Animal Care and Use Committee of Chonbuk National University. The animals were randomly assigned to the following four groups of ten mice each: adeno-virus diluted in sterile 0.9% NaCl and injected intravenously (i.v.) through the tail vein in the control group (without DSS; Ade-cont), COMP-Ang1-virus injected control group (without DSS; comp-cont), adeno-virus injected 3% DSS administration group (Ade-DSS), and COMP-Ang1-virus injected 3% DSS administration group (comp-DSS). The colitis model was induced in mice by adding filtered 3% DSS to drinking water for 7 days. (DSS, molecular weight 36–50 kDa; MP Biochemicals, Aurora, OH, USA); mice in the control group received tap water without DSS. Mice were monitored daily for body weight, symptom of stool, fecal occult blood, and survival [17]. The disease activity index (DAI) was determined on the basis of mean scores of weight change, stool parameters, and fecal occult blood, as described previously [18, 19].

### Histopathological analysis

The colon was fixed using 4% paraformaldehyde and embedded in paraffin. Five-micrometer-thick sections were sliced from the paraffin block and stained with hematoxylin and eosin (H & E). Colon damage was assessed as previously described. The evaluation parameters were extent of injury, wall edema, leukocyte infiltration, and crypt abscesses [20]. The degree of inflammation was scored as follows: on a scale of 0–3 (0, negative; 1, mild; 2, moderate; 3, severe), as was the extent of injury (0, negative; 1, mucosal; 2, mucosal and muscularis mucosal; 3, transmural); damage in crypt architecture was scored as follows: on a scale of 0–4 (0, negative; 1, 0–30% damage to epithelium; 2, 31–65% damage to epithelium; 3, structurally defective epithelium; 4, loss of crypt and epithelium destruction). Each section was graded on the basis of affect the mount of involvement on a scale of 1–4 (1, 0–25%; 2, 26–50%; 3, 51–75%; 4, 76–100%). At least five sections from slide were examined to derive each score. The scoring system was designed to yield a minimum of 0 and a maximum of 40.

### Immunohistochemistry

Colon sections were fixed using 4% paraformaldehyde and embedded in paraffin. The paraffin block was cut into 4 μm sections, deparaffinized with xylene and rehydrated with ethanol. After blocking for 1 h, the colon tissue was incubated overnight at 4 °C with anti-mouse lymphatic vessel endothelial hyaluronan receptor 1 (LYVE-1) (Angiobio, Del Mar, CA, USA), anti-vascular endothelial growth factor receptor-3 (VEGFR-3) (R & D systems, Minneapolis, MN, USA), and anti-ER-HR3 antibodies (BMA, Augst, Switzerland). The sections were treated with AEC substrate-chromogen (DakoCytomation, Glostrup, Denmark) to visualize the immunocomplexes. Immunohistochemical staining was visualized under a Nikon Eclipse 80*i* light microscope (Nikon Instruments Inc., Melville, NY, USA). The densities of LYVE-1-positive and VEGFR-3-positive areas were measured in 12 randomly selected fields at a magnification of ×400 using the ImageJ software.

### Quantitative real-time PCR

Total RNA from the colon was isolated using the RNeasy mini kit (Qiagen, Hilden, Germany), and the first strand of cDNA was synthesized using a Transcriptor First Strand cDNA synthesis kit (Roche, Mannheim, Germany). Real-time qPCR was performed using iTaq universal SYBR Green Supermix (Bio-Rad, Hercules, CA, USA) according to the manufacturer’s protocol. Primers for all the genes were as follows. VEGF-A sense: 5′- GCT GTA CCT CCA CCA TGC CAA C-3′; VEGF-A antisense: 5′- CGC ACT CCA GGG CTT CAT CG-3′; VEGF-C sense: 5′- AGA CGG ACA CAC ATG GAG GT-3′; VEGF-C antisense: 5′- AAA GAC TCA ATG CAT GCC AC-3′; VEGF-D sense: 5′- TTG AGC GAT CAT CCC GGT C-3′; VEGF-D antisense: 5′- GCG TGA GTC CAT AGG GCA A-3′; IL-1β antisense: 5′-TCT TCT TTG GGT ATT GCT TGG-3′; IL-6 sense: 5′-TGG AGT ACC ATA GCT ACC TGG A-3′; IL-6 antisense: 5′-TGA CTC CAG CTT ATC TGT TAG GAG-3′; TNF-α sense: 5′-ACC CTC ACA CTC AGA TCA TC-3′; TNF-α antisense: 5′-GAG TAG ACA AGG TAC AAC CC-3′; CD80 sense: 5′-GGCAAGGCAGCAATACCTTA-3′; CD80 antisense: 5′-CTCTTTGTGCTGCTGATTCG-3′; iNOS sense: 5′- TTCTGTGCTGTCCCAGTGAG-3′; iNOS antisense: 5′-TGAAGAAAACCCCTTGTGCT-3′; CD206 sense: 5′-CAAGGAAGGTTGGCATTTGT-3′; CD206 antisense: 5′-CCTTTCAGTCCTTTGCAAGC-3′; Arg-1 sense: 5′-CAGAAGAATGGAAGAGTCAG-3′; Arg-1 antisense: 5′-CAGATATGCAGGGAGTCACC-3′; GAPDH sense: 5′- TTG ATG GCA ACA ATC TCC AC -3′; GAPDH antisense: 5′- CGT CCC GTA GAC AAA ATG GT -3′.

### Enzyme-linked immunosorbent assay

The levels of IL-1β and IL-6 in colon tissue were measured using a DuoSet sandwich ELISA kit (Enzo Life Sciences, Farmingdale, NY, USA).

### Statistical analysis

Data are expressed as the means ± SD. Mean comparisons between two groups were examined for significant differences using analysis of variance (ANOVA), followed by individual comparisons using a Tukey’s post hoc test; *P* value < 0.05 was considered to indicate a statistically significant difference.

## Results

### Inflammation-induced lymphangiogenesis in experimental colitis

**Fig. 1:**
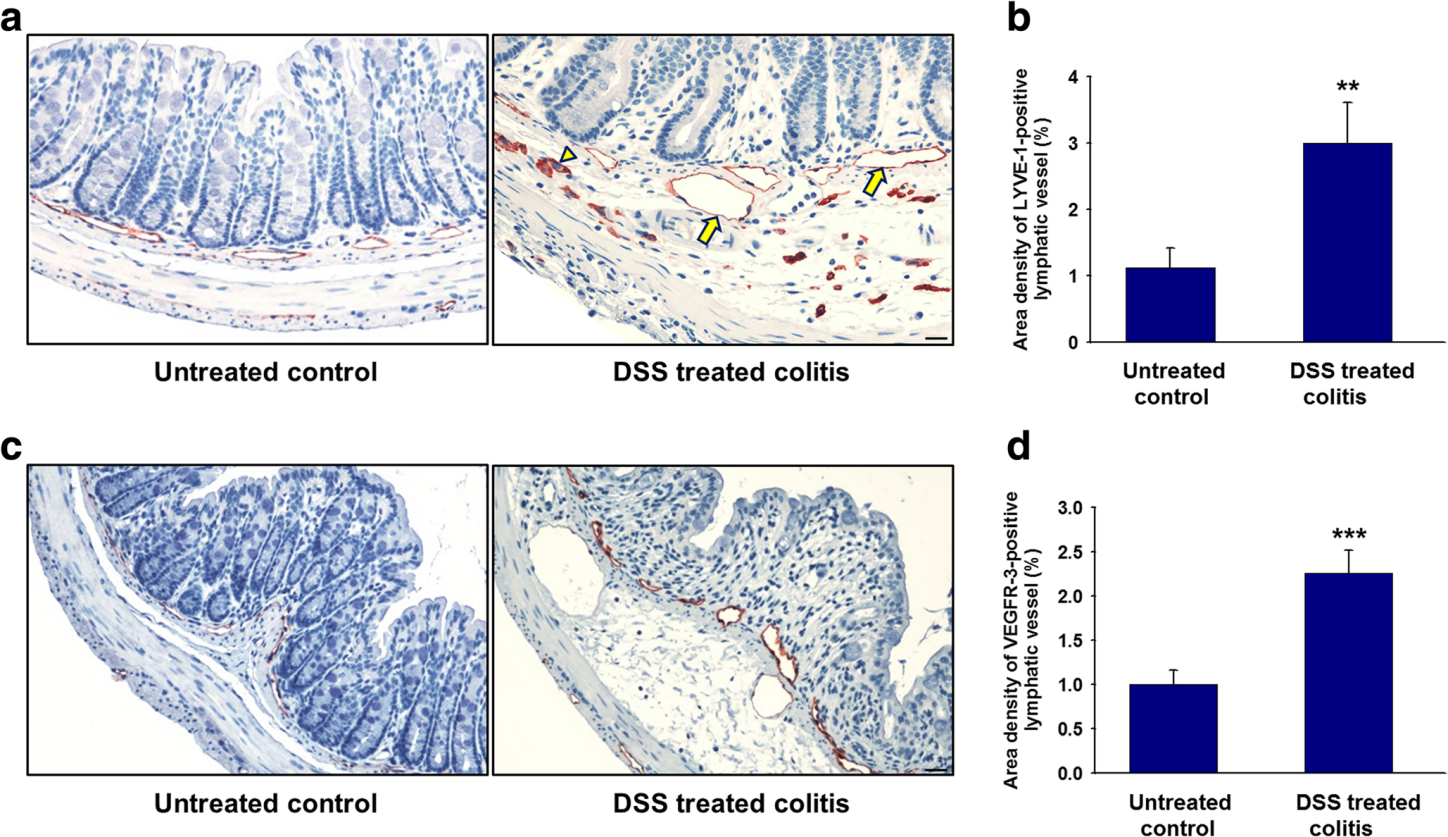
Lymphatic density is increased in the colons of mice with DSS-induced colitis. **a** Immunohistochemical staining with an antibody recognizing LYVE-1 and **c** VEGFR-3 were performed on untreated control and DSS-treated colitis model. LYVE-1-positive lymphatic vessels (indicated by yellow arrow) and LYVE-1-expressing monocytes were also observed (indicated by yellow arrow head, Fig. 1a) Quantitative analysis of lymphatic vessel density in the colon sections is shown in **b** and **c**. LYVE-1-positive and VEGFR-3-positive lymphatic vessels were quantified by measuring ten regions per section; non-overlapping fields at ×40 magnification were quantified (*n* = 10/group). Data are expressed as the means ± SD. Scale bar 50 μm. **P < 0.01 vs. untreated control; ***P < 0.001 vs. untreated control

Inflammation remodels the lymphatic network by a process known as lymphangiogenesis. During disease onset, intestinal lymphatics are generated from the existing vessels and extremely dilated lymphatic vessels in DSS-induced experimental colitis mice. We observed that the LYVE-1-positive lymphatic vessels were distributed in the submucosal layers of the colon in untreated control mice, whereas the DSS-induced mice had enlarged LYVE-1-positive lymphatic vessels with a significantly higher density compared to the untreated mice (Fig. 1a, b). In addition, LYVE-1-expressing monocytes were also observed (indicated by yellow arrow head, Fig. 1a). Similarly, the levels of VEGFR-3, a representative lymphatic marker, were also increased in the inflamed colon (Fig. 1c, d).

### Systemic delivery of COMP-Ang1 ameliorates body weight loss, DAI, and colon shortening in mice with colitis

**Fig. 2:**
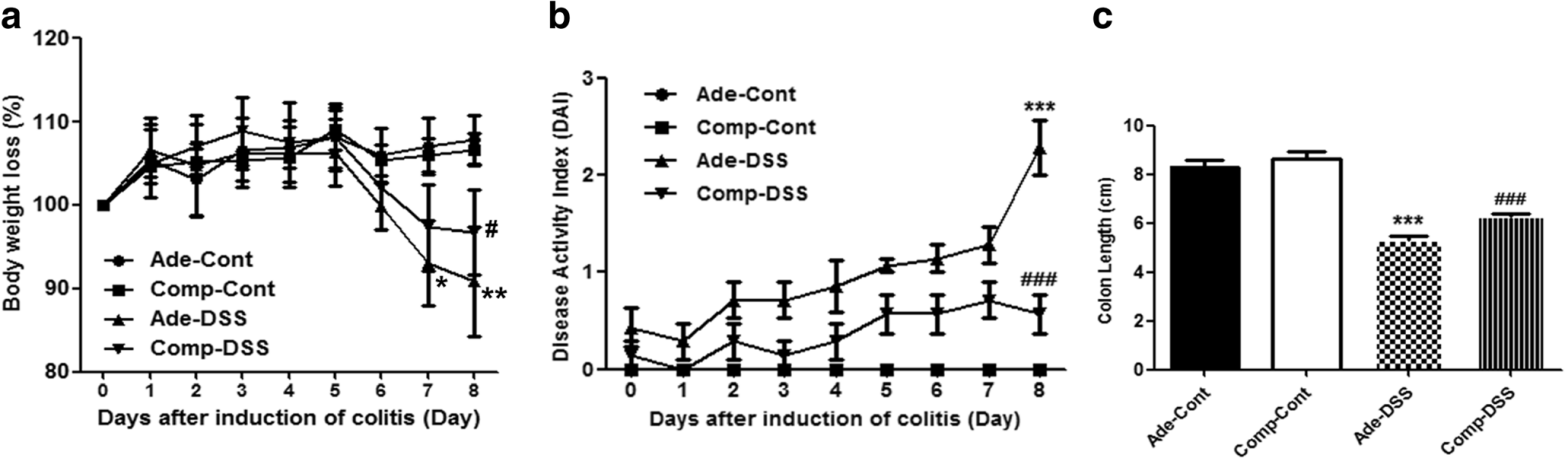
Systemic delivery of COMP-Ang1 ameliorates body weight loss, disease activity index (DAI), and colon shortening in mice with DSS-induced colitis. **a** Level of body weight loss(%) and **b** DAI scores. **c** Colon lengths were measured 0–7 days after initiating DSS administration. Data shown are from three independent experiments and are expressed as means ± SD (*n* = 10 per group). ***P < 0.001 vs. Ade-Cont; ###*P* < 0.001 vs. Ade-DSS

We examined signs of disease such as body weight loss, DAI, and colitis-induced shortening of the colon 7 days after administration of 3% DSS-supplemented drinking water. We observed that the body weight in Ade-DSS-treated mice and COMP-DSS-treated mice showed no significant difference (Fig. 2a). However, the DAI was markedly decreased in the COMP-DSS-treated mice compared to the Ade-DSS-treated mice (Fig. 2b). COMP-treatment recovered colon length in COMP-DSS-treated mice by day 7 (Fig. 2c).

### COMP-Ang1 decreases inflammation-induced lymphangiogenesis in a DSS-induced colitis model

**Fig. 3:**
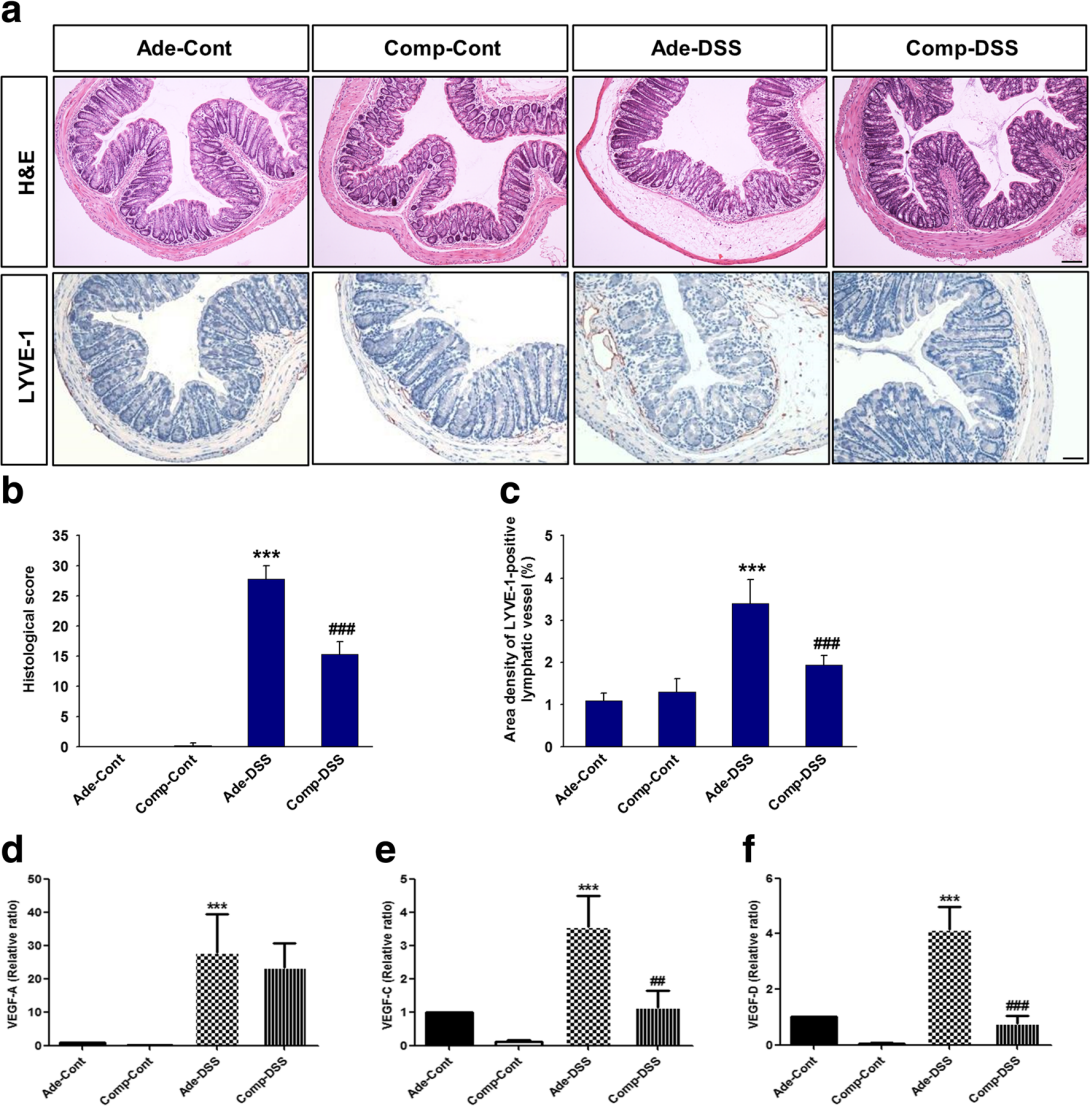
COMP-Ang1 decreases inflammation-induced lymphangiogenesis in a DSS-induced colitis model. **a** Representative hematoxylin and eosin staining and LYVE-1 immunohistochemistry images of colons from the indicated groups of mice. Scale bar 10 μm. **b** Histopathological scores of the analyzed slides. **c** Quantitative analysis of LYVE-1-positive lymphatic vessel density in the colon. Expression of **d** VEGF-A, **e** VEGF-C, and **f** VEGF-D levels was determined by quantitative real-time PCR. The expression of these genes was normalized to that of GAPDH. Bars represent the means ± SD of three independent experiments. ***P < 0.001 vs. Ade-Cont; ##*P* < 0.01 vs. Ade-DSS; ###*P* < 0.001 vs. Ade-DSS

Recent studies showed that lymphatic vessels (LVs) are functionally important for the resolution of inflammatory response [1]. To investigate the changes in lymphatic density in the DSS-induced colitis model, we performed immunohistochemical staining for LYVE-1, a marker of lymphatic vessel endothelial cells. The colon sections of Ade-Cont-treated mice showed only a narrow alley and thin LVs in the lamina propria and submucosa (Fig. 3a). However, after 7 days of DSS treatment, the mucosa was inflamed in Ade-DSS-treated mice, accompanied by increase in the density of enlarged LYVE-1-positive lymphatic vessels and colon submucosa edema, indicating decreased lymphatic vessel function. Surprisingly, systemic delivery of COMP-Ang1 significantly reduced the density of lymphatic vessels in the DSS-induced colitis model (Fig. 3a, c). The Ade-DSS-induced inflammatory changes that progressed to multifocal erosions, crypt loss, infiltration of leukocytes, and increased submucosa edema were improved by COMP-Ang1 treatment (Fig. 3a, b).

### COMP-Ang1 reduces the expression of VEGF-A, VEGF-C, and VEGF-D in mice with colitis

Reports show that inflammation-induced lymphangiogenesis correlates with expression of the VEGF family members. We quantified the mRNA to determine the expression of level of VEGF family members in the colon tissue. Results showed that the expression levels of VEGF-A (20.5 fold), VEGF-C (3.4 fold), and VEGF-D (3.9 fold) were increased in Ade-DSS mice, whereas those of VEGF-C and VEGF-D were markedly suppressed in COMP-DSS mice (Fig. 3d–f). VEGF-A expression was decreased in COMP-DSS mice compared to that in Ade-DSS mice, although it was not statistically significant (Fig. 3d).

### COMP-Ang1 reduces the expression of pro-inflammatory cytokines in a DSS-induced colitis model

**Fig. 4:**
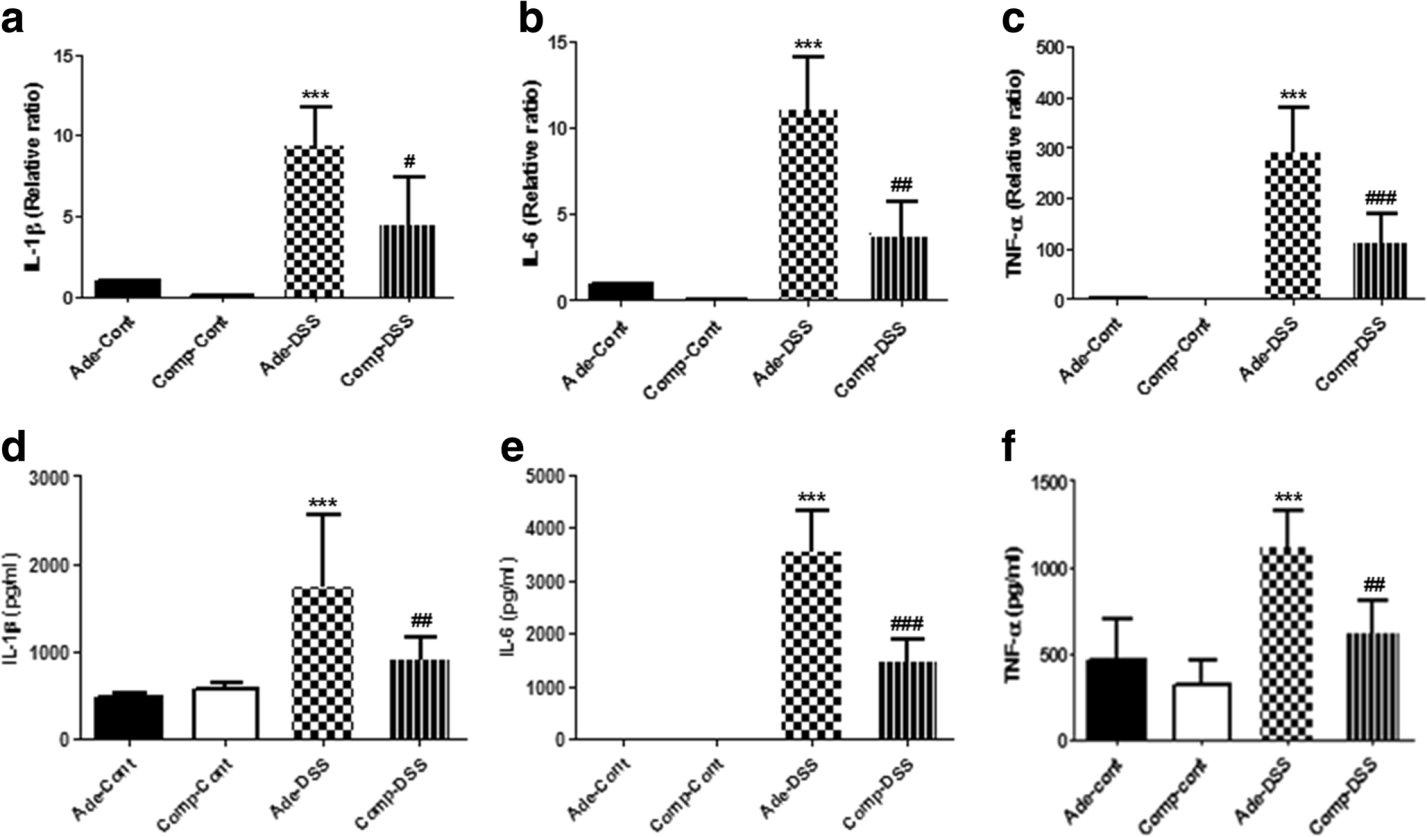
COMP-Ang1 reduces the expression of pro-inflammatory cytokines in a DSS-induced colitis model. Colonic expression of pro-inflammatory cytokines. **a** IL-1β, **b** IL-6, and **c** TNF-α were examined by real-time PCR. Protein levels of **d** IL-1β **e** IL-6, and **f** TNF-α were measured using ELISA. Data represent the means ± SD of three independent experiments. ***P < 0.001 vs. Ade-Cont; #*P* < 0.05 vs. Ade-DSS; ##*P* < 0.01 vs. Ade-DSS; ###*P* < 0.001 vs. Ade-DSS

Secreted pro-inflammatory cytokines may cause inflammation-induced lymphangiogenesis in mice with colitis. To investigate changes in pro-inflammatory cytokine levels in a DSS-induced colitis model, we measured IL-1β, IL-6, and TNF-α levels at the mRNA (RT-PCR) and protein (ELISA) levels in the colon. Quantitative real-time PCR (qPCR) analysis showed that the upregulation of IL-1β, IL-6, and TNF-α in Ade-DSS mice was significantly decreased in COMP-DSS mice (Fig. 4a–c). In addition, ELISA showed the same trend for IL-1β, IL-6, and TNF-α levels (Fig. 4d–f).

### COMP-Ang1 prevents infiltration of macrophages in a DSS-induced colitis model

**Fig. 5:**
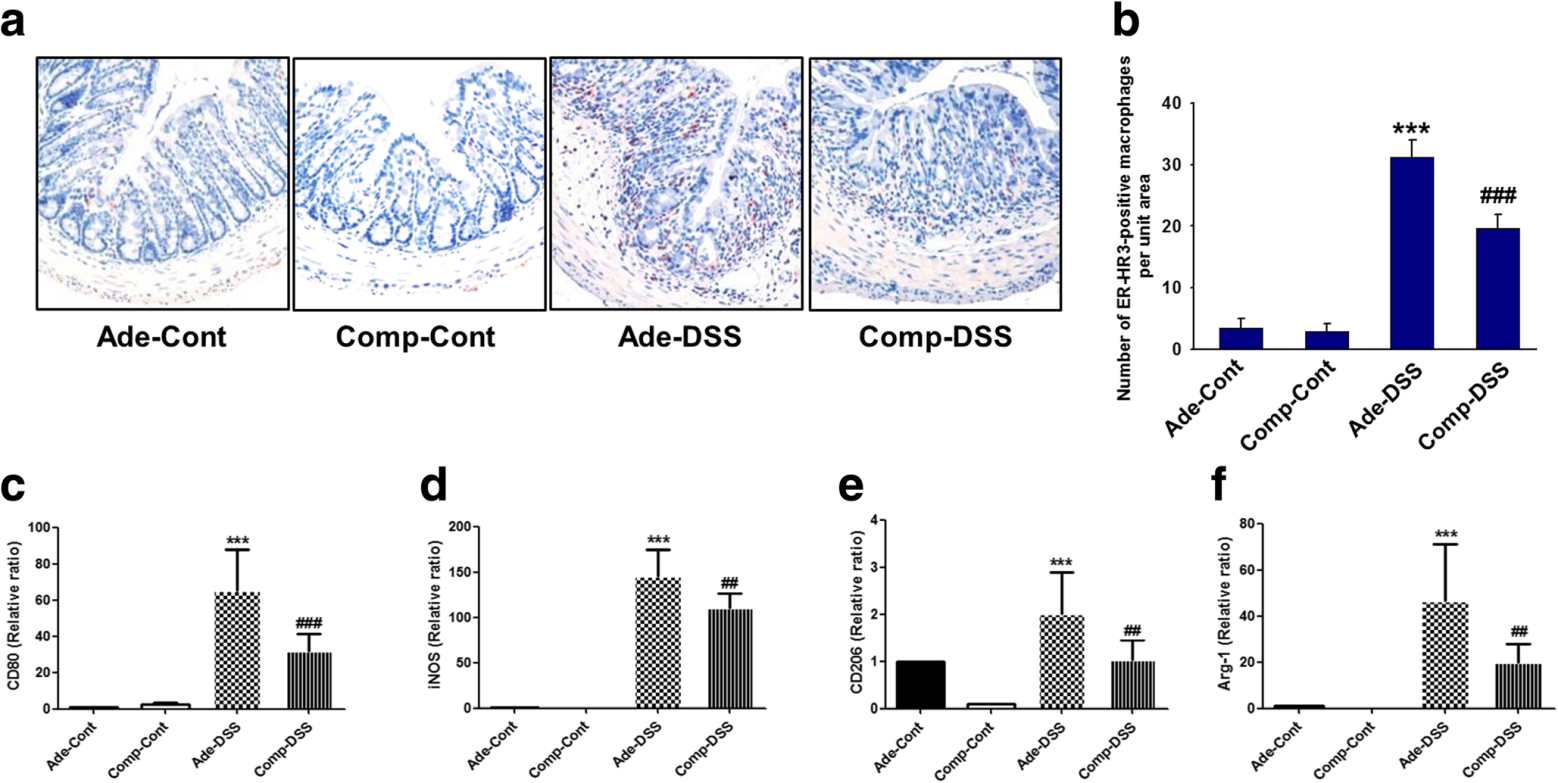
COMP-Ang1 prevents macrophage infiltration in a DSS-induced colitis model. **a** Representative images of paraffin-embedded sections from mice with DSS-induced colitis were stained with anti-ER-HR-3, and the number of ER-HR3-positive macrophages was quantified in each group. Scale bar 10 μm. **b** The expression levels of macrophage-associated genes, **c** CD80, **d** iNOS, **e** CD206, and **f** Arg-1, were examined by real-time PCR. The expression of these genes was normalized to that of GAPDH. Data represent the means ± SD of three independent experiments. **P < 0.01 vs. Ade-Cont; ***P < 0.001 vs. Ade-Cont; ##*P* < 0.01 vs. Ade-DSS; ###*P* < 0.001 vs. Ade-DSS

Activated macrophages may have critical roles in the regulation of inflammatory processes. Inflammation-induced lymphangiogenesis and the involvement with polarized that classically activated (M1) and alternatively activated (M2) macrophages have considerably improved in recent years. We measured the number of ER-HR3-positive macrophages in the inflamed colon by immunohistochemistry to further examine the inhibitory effects of COMP-Ang1 on infiltration of inflammatory macrophages (Fig. 5a, b). Treatment with COMP-Ang1 reduced the DSS-induced increase in infiltration of ER-HR3-positive macrophages in colitis tissue. Next, we evaluated the expression of M1 and M2 macrophage-related factors after treatment with INF-γ and IL-4, respectively; qPCR was used to investigate iNOS and CD80 levels as M1 macrophage-related factors, whereas arginase-1 and CD206 were investigated as M2 macrophage-related factors. Compared to Ade-DSS, COMP-Ang1 significantly decreased the expression of both M1 and M2 macrophage-related factors (Fig. 5c–f).

## Discussion

DSS administration induces typical sign of colitis such as DAI increase, body weight loss, and a shortening of the colon [21]. Presence of occult blood and diarrhea are usually the earliest features, and the inflammation fully develops within 7–10 days. Macroscopic features include shortened edematous colon with areas of hemorrhage and ulceration in H & E staining. Infiltration of inflammatory cells, such as macrophages, plasma cells, and few lymphocytes was observed in the mucosa and submucosa. Mucosal edema, goblet cell loss, and crypt destruction were followed by crypt shortening [21]. COMP-Ang1 administration alleviated these symptoms, which indicated that COMP-Ang1 reduced DSS-induced colitis. Furthermore, COMP-Ang1 may possess a therapeutic range in which it exerts both anti-inflammatory and beneficial effects in colitis.

IBD is a complex process involving most type of immune cells of the microvasculature. It causes chronic inflammation via leukocyte recruitment, angiogenesis, and lymphangiogenesis, which results in tissue remodeling. IBD is the result of dysfunctional immunoregulation which is evident by the production of mucosal cytokines that contribute to increase in blood and lymphatic vessel density in IBD [22]. However, the precise mechanism of inflammation-induced lymphangiogenesis is still unknown. Ran et al. [23] asserted that induction of the NF-κβ pathway by inflammatory stimuli activates the transcription factor Prox-1 (Prospero homeobox protein 1), which is a specific marker of the lymphatic endothelium. NF-κβ and Prox-1 activate the VEGFR-3 promoter and enhance the response of the lymphatic endothelium to the VEGFR-3 binding factors, VEGF-C and VEGF-D [24]. Reports show that lymphatic vessel density and VEGF-C/VEGFR-3 signaling are increased in the colon of IBD patients [25].

Lymphangiogenesis is occasionally present during inflammation [26]. Lymphangiogenesis with inflammatory conditions, including colitis, is involved in the physiology of inflammation. It may directly influence mucosal edema or immune cell infiltration in inflamed tissues [3]. During inflammation, inflammatory mediators pass through the LVs, which play an important role in maintaining fluid homeostasis by absorbing tissue fluid [27]. Inflammation-induced lymphangiogenesis correlates with the expression of VEGF family members, because of the lymphangiogenic role of CD11b macrophage [28]. Infiltrated macrophages expressing VEGF-C and VEGF-D are observed during intestinal inflammation [29, 30], which might contribute to inflammation-induced lymphangiogenesis. COMP-Ang1 treatment reduced the increase in the number of ER-HR3-positive macrophages infiltrating the kidney in ischemia-reperfusion-induced renal injury. [31] In this study, COMP-Ang1 drastically diminished the levels of inflammatory cytokines and reduced macrophage infiltration in DSS-induced colitis. Therefore, COMP-Ang1 may be considered a candidate for anti-inflammatory therapy to reduce inflammation-induced lymphangiogenesis-related inflammatory cytokine levels and the number of infiltrated macrophages.

When macrophages are activated, M1 and M2 types of macrophages elicit different responses. Recently, it has been reported that the mechanism of M1 or M2 polarization may regulate VEGF production by macrophages [32]. Indeed, the expression of VEGF-C is increased in both M1 and M2 type macrophages in obstructed renal inflammation [33]. Our results showed that COMP-Ang1 decreased the expression of CD80, iNOS, CD206, and Arg-1-related genes of macrophage polarization, and VEGF-C and VEGF-D. COMP-Ang1 also affected macrophage polarization, leading to decrease in VEGF-C production.

IBD pathogenesis involves impaired clearance of foreign material, leading to sustained activation of innate immune cells and compensatory induction of the adaptive immune response [34]. Although not assessed in our study, it would be of interest to investigate the in vivo effects of COMP-Ang1, such as systemic elimination of macrophages or antigen clearance for epithelial barrier of colon.

In conclusion, our observations support the use of COMP-Ang1 as a novel therapeutic that might reduce inflammation-induced lymphangiogenesis in IBD and other inflammatory diseases.

## Electronic supplementary material

Supplementary Figure 1 COMP-Ang1 inhibits expression of intercellular adhesion molecule-1 (ICAM-1) in a DSS-induced colitis model. **a** Representative images of paraffin-embedded sections from DSS-induced colitis mice were stained with anti-ICAM-1. The density of ICAM-1 expression was quantified in each group. Scale bar: 50 μm. **b** Quantitative analysis of density of ICAM-1 (%) in the colon. Data represent the means ± SD of three independent experiments. ***, *P* < 0.001 vs. Ade-Cont; ##, *P* < 0.01 vs. Ade-DSS (GIF 215 kb)

High resolution image (TIFF 4197 kb)

Supplementary Figure 2 Expression of Arginase-1 and F4/80 in macrophages in during colitis. **a** Tissues were fixed in 4% formaldehyde solution, and then frozen sections were stained with anti-Arg-1 and anti-F4/80 antibodies. Nuclei were stained with DAPI (4′,6-diamidino-2-phenylindole; blue color). Note that Arg-1 was cellular expressed in the F4/80-positive cells of the colon. Scale bar: 5 μm. **b** Quantitative analysis of the number of Arg-1 and F4/80-positive macrophages in the colon. Data represent the means ± SD of three independent experiments. **, P < 0.01 vs. Ade-Cont; #, *P* < 0.05 vs. Ade-DSS (GIF 66 kb)

High resolution image (TIFF 1262 kb)
