# Supplementary figures and images for: COMP-angiopoietin-1 ameliorates inflammation-induced lymphangiogenesis in dextran sulfate sodium (DSS)-induced colitis model
Source: J Mol Med (Berl). 2018 Apr 2;96(5):459–67. doi: 10.1007/s00109-018-1633-x (PMC5897474; doi:10.1007/s00109-018-1633-x)

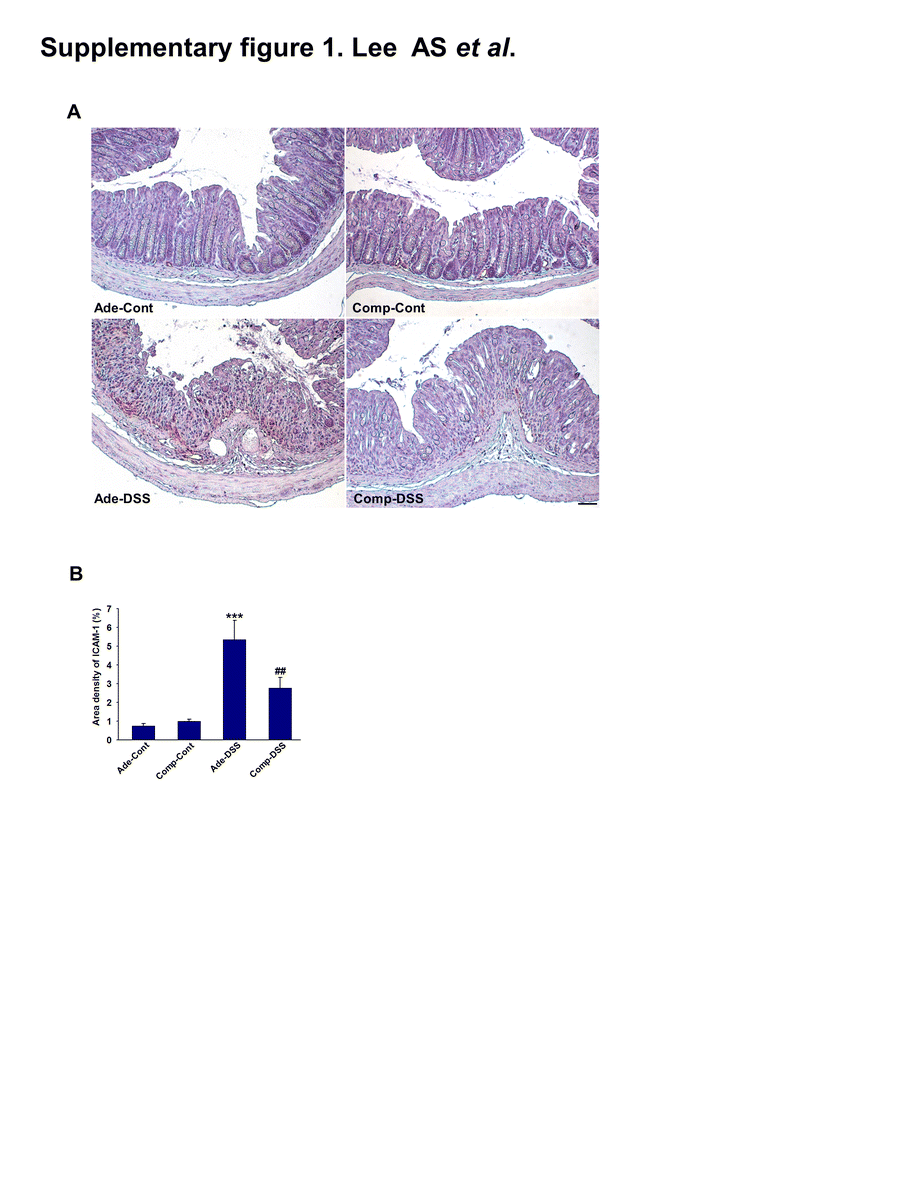

Supplement: Supplementary file 1 — COMP-Ang1 inhibits expression of intercellular adhesion molecule-1 (ICAM-1) in a DSS-induced colitis model. a Representative images of paraffin-embedded sections from DSS-induced colitis mice were stained with anti-ICAM-1. The density of ICAM-1 expression was quantified in each group. Scale bar: 50 μm. b Quantitative analysis of density of ICAM-1 (%) in the colon. Data represent the means ± SD of three independent experiments. ***, P < 0.001 vs. Ade-Cont; ##, P < 0.01 vs. Ade-DSS (GIF 215 kb) [file 109_2018_1633_Fig6_ESM.gif]

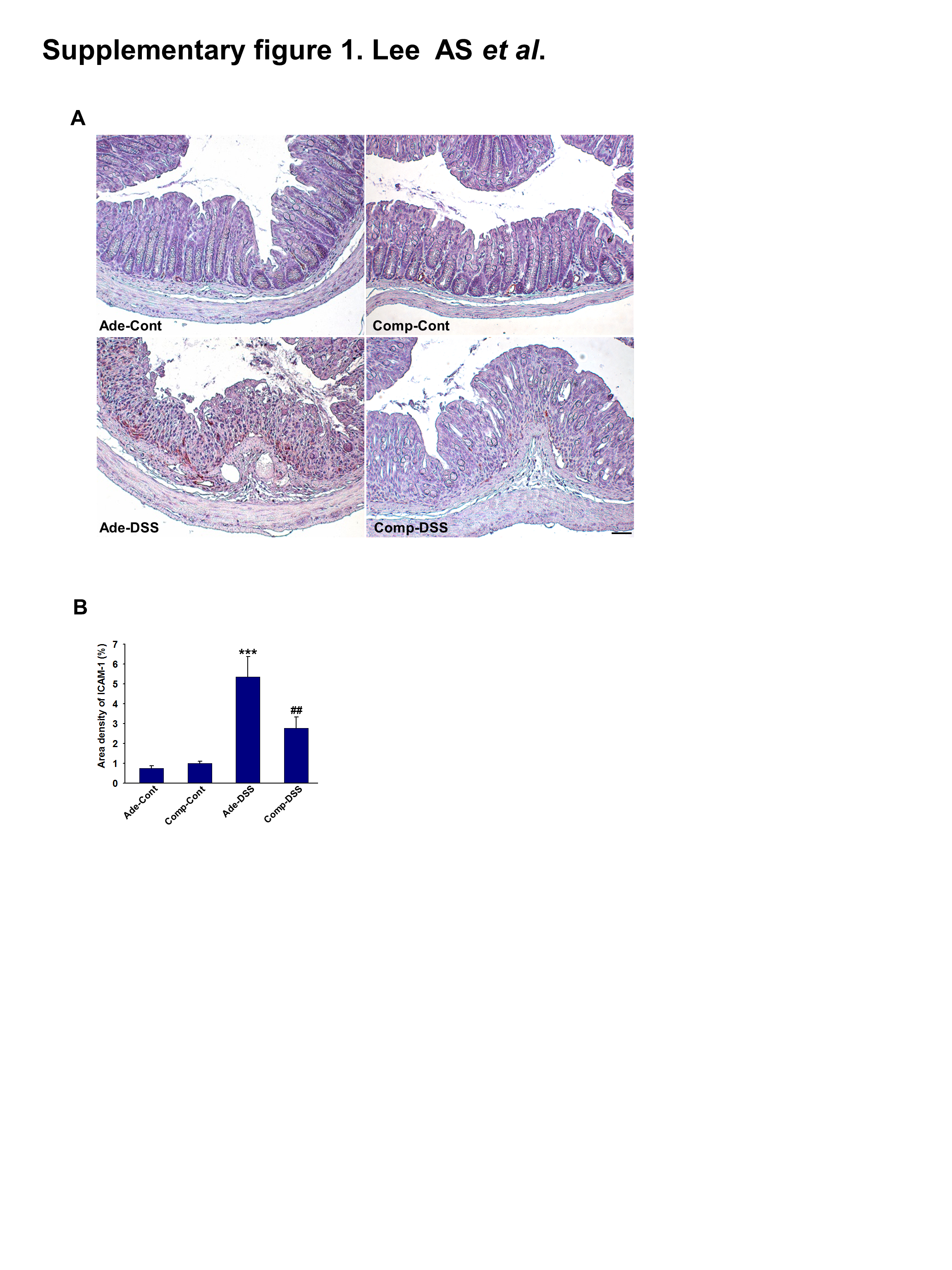

Supplement: Supplementary file 2 — High resolution image (TIFF 4197 kb) [file 109_2018_1633_MOESM1_ESM.tif]

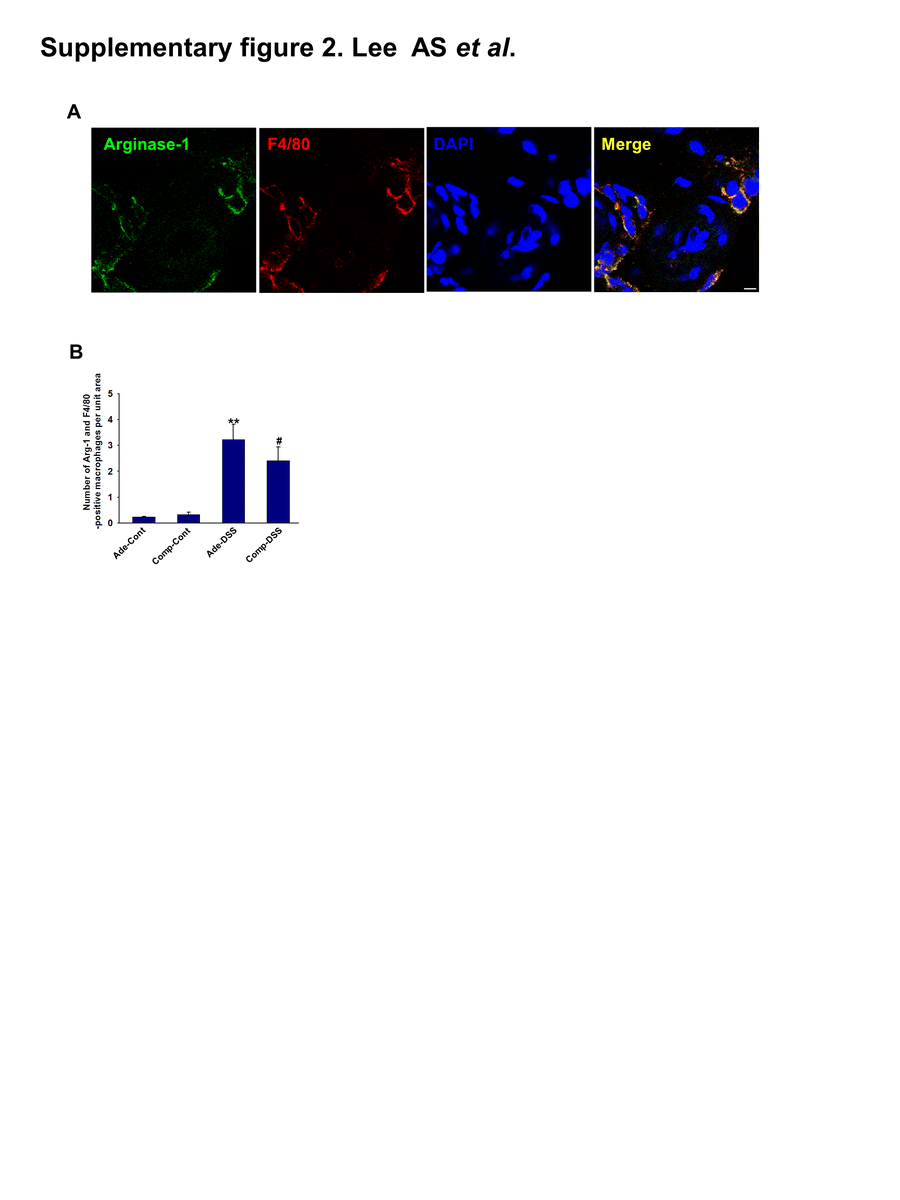

Supplement: Supplementary file 3 — Expression of Arginase-1 and F4/80 in macrophages in during colitis. a Tissues were fixed in 4% formaldehyde solution, and then frozen sections were stained with anti-Arg-1 and anti-F4/80 antibodies. Nuclei were stained with DAPI (4′,6-diamidino-2-phenylindole; blue color). Note that Arg-1 was cellular expressed in the F4/80-positive cells of the colon. Scale bar: 5 μm. b Quantitative analysis of the number of Arg-1 and F4/80-positive macrophages in the colon. Data represent the means ± SD of three independent experiments. **, P < 0.01 vs. Ade-Cont; #, P < 0.05 vs. Ade-DSS (GIF 66 kb) [file 109_2018_1633_Fig7_ESM.gif]

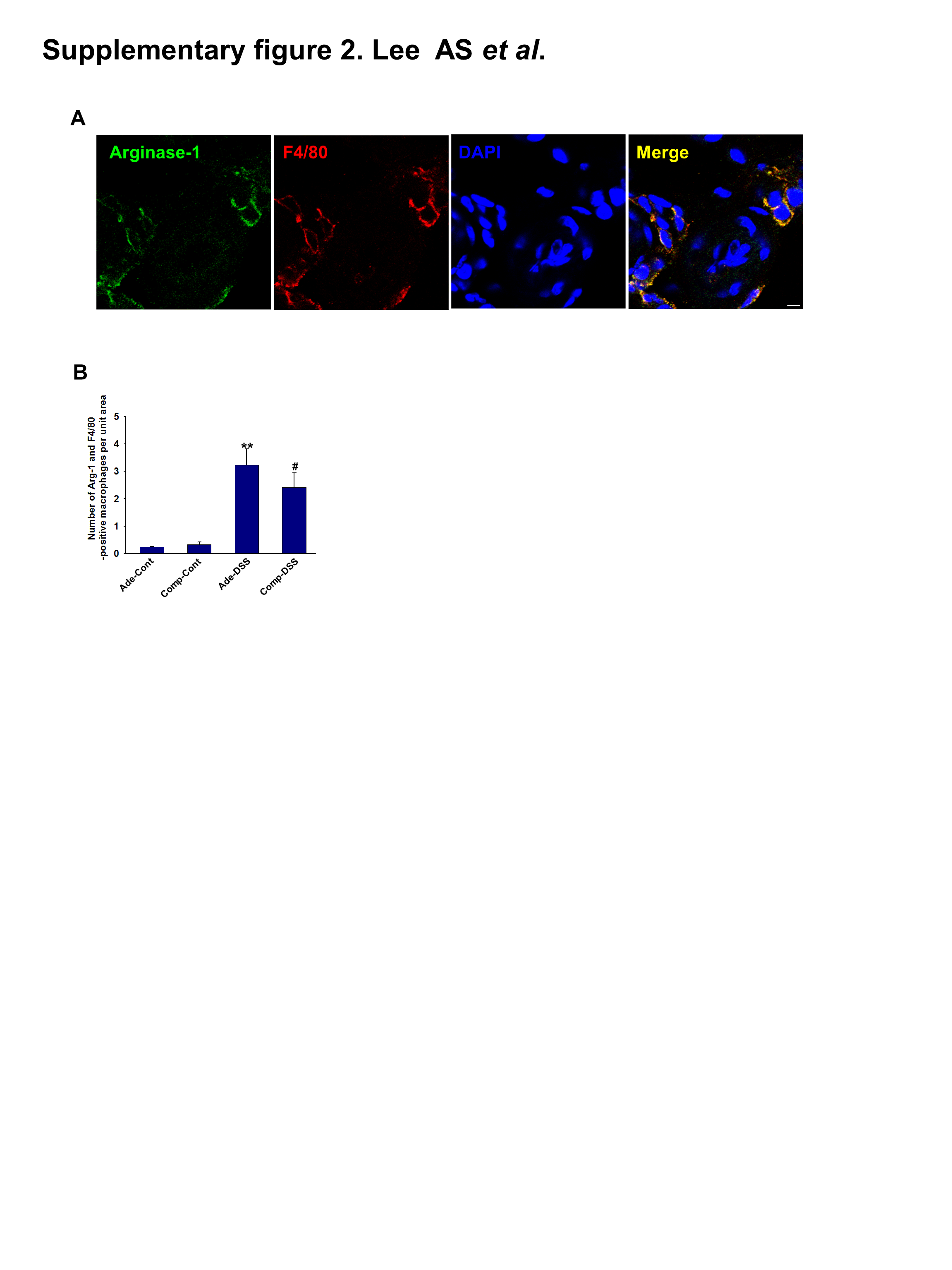

Supplement: Supplementary file 4 — High resolution image (TIFF 1262 kb) [file 109_2018_1633_MOESM2_ESM.tif]
